# Correlative Light and Transmission Electron Microscopy Showed Details of Mitophagy by Mitochondria Quality Control in Propionic Acid Treated SH-SY5Y Cell

**DOI:** 10.3390/ma13194336

**Published:** 2020-09-29

**Authors:** Minkyo Jung, Hyosun Choi, Jaekwang Kim, Ji Young Mun

**Affiliations:** 1 Neural Circuit Research Group, Korea Brain Research Institute, Daegu 41062, Korea; j0312@kbri.re.kr (M.J.); hyokchoi0123@gmail.com (H.C.); 2 BK21 Plus Program, Department of Senior Healthcare, Graduate School, Eulji University, Daejeon 34824, Korea; 3 Dementia Research Group, Korea Brain Research Institute, Daegu 41062, Korea; kim_jaekwang@kbri.re.kr

**Keywords:** propionic acid, autophagy, mitophagy, correlative light and electron microscopy (CLEM)

## Abstract

Propionic acid is a metabolite of the microbiome and can be transported to the brain. Previous data show that propionic acid changes mitochondrial biogenesis in SH-SY5Y cells and induces abnormal autophagy in primary hippocampal neurons. Maintaining mitochondrial function is key to homeostasis in neuronal cells, and mitophagy is the selective autophagy involved in regulating mitochondrial quality. Monitoring mitophagy though light microscopy or conventional transmission electron microscopy separately is insufficient because phases of mitophagy, including autophagosome and autolysosome in nano-resolution, are critical for studies of function. Therefore, we used correlative light and electron microscopy to investigate mitochondrial quality in SH-SY5Y cells after propionic acid treatment to use the advantages of both techniques. We showed, with this approach, that propionic acid induces mitophagy associated with mitochondrial quality.

## 1. Introduction

Short-chain fatty acids (SCFAs) such as acetic, propionic, and butyric acid are by-products of fermentation of dietary fiber by the gut microbiome [1]. Microbe-derived metabolites can cross the blood–brain barrier and affect the neurons. As the relationship between gut microbiome and the brain, gut–brain axis, has become interesting, SCFAs have attracted increasing attention. Propionic acid (PPA) is increased in stools from patients with autistic spectrum disorder, and prenatal exposure to PPA causes significant impairment of the social behavior of neonatal rat offspring [2]. Further, PPA administration to rodents alters expression of genes associated with neurotransmitters, neuronal cell adhesion molecules, inflammation, oxidative stress, lipid metabolism, and mitochondrial function [3,4,5]. Conversely, decreases in PPA are reported in patients with multiple sclerosis, an autoimmune and neurodegenerative disease [6]. One important cellular process negatively affected by PPA is mitochondrial function [7]. Rats exposed to PPA show mitochondrial dysfunction and an increase in free acyl-carnitine, a factor for the transport of long-chain fatty acids into mitochondria [8]. PPA and butyric acid also induce autophagy in human colon cancer cells that limits apoptosis, and inhibition of autophagy potentiates SCFA-induced apoptosis [9]. As our previous data indicated that PPA induces abnormal autophagy in PPA-treated hippocampal neuron [10], we investigated the relationship between mitochondrial defects and the regulation of mitochondrial quality though mitophagy.

Mitophagy is a selective degradative process responsible for removing damaged mitochondria to maintain cytoplasmic homeostasis [11]. Mitochondrial dysfunction is involved in various neurodegenerative or neurodevelopmental diseases [12]. Once mitophagy is initiated, a balance between autophagosome formation and autophagic degradation is necessary. Thus, accumulation of autophagosomes and disruption of the autophagic process in neurons is associated with disease [13]. Until now, conventional techniques for analysis of mitophagy have been based on immunofluorescence staining and immunoblotting of several specific mitochondrial proteins, qPCR for mitochondrial DNA copy number, and nano-resolution imaging using transmission electron microscopy (TEM). TEM is a direct imaging method for the early stage of mitophagy, which is the starting point of engulfing mitochondria and early autophagosome showing specific mitochondrial structures such as cristae [14,15]. However, the assessment of the late phases of mitophagy requires specific imaging techniques.

Recently, the engineering of two fluorescent proteins (mCherry-GFP-mito, and mt-Keima) has permitted monitoring of the status of mitophagy in live cells. These reporters change the fluorescence profile in response to pH changes. For example, the excitation wavelength for mt-Keima is 488 at neutral pH and 561 at acidic pH for late mitophagy observed in the lysosome [16,17]. mCherry-GFP-mito protein, fused to a mitochondrial targeting sequence of a mitochondrial protein, such as the outer mitochondrial membrane (OMM) protein FIS1 (comprising amino acids 101–152) [18] can be used to detect mitophagy. The mitochondrial network can be seen as a green fluorescence, and mitochondria delivered to lysosomes show as a red color after mitophagy. This is because mCherry fluorescence is stable, but GFP fluorescence is quenched in the acidic condition. However, these tools do not allow the monitoring of all phases of mitophagy. To analyze the entire dynamic phase of autophagy regarding mitophagy, TEM is employed to classify the specific type of autophagy including phagophore, autophagosome, and autolysosome in high resolution [19,20]. Therefore, we analyzed structural changes by TEM to study specific stages of autophagy that are more tightly linked with the mechanisms of mitophagy dysfunction. Thus, correlative light and electron microscopy (CLEM) is an effective method to analyze mitophagy or autophagic pathways [21]. Because the Keima protein is incompatible with fixation [22], we used GFP and mCherry conversion depending on the pH level of lysosomes to investigate details of various steps of mitophagy in nano-resolution though electron microscopy (EM). The CLEM technique of overlaying two images from fluorescence and EM makes the investigation of all phases of mitophagy possible. Evans et al. suggested that CLEM can open new avenues using light-up through (fluorescent) dyes in the dark by EM observation [23]. Thus, we applied CLEM to study mitophagy after PPA treatment.

## 2. Materials and Methods

### 2.1. Cell Culture

SH-SY5Y control cells, obtained from Dr. Kim H.J (KBRI), and the tandem mCherry–GFP tag fused to FIS1 stable SH-SY5Y cells, a kind gift from Dr. Ian G. Ganley (University of Dundee, Dundee, UK) [18], were grown in normal culture conditions with DMEM/F12 (ThermoFisher, Waltham, MA, USA) supplemented with 15% fetal bovine serum (ThermoFisher, USA), 100 units/mL of penicillin, and 100 ug/mL of streptomycin (ThermoFisher, USA) at 37 °C in a humidified 5% CO_2_ atmosphere. The tandem mCherry–GFP tag fused to FIS1 stable SH-SY5Y cells were selected with 500 µg/mL of hygromycin (Sigma, St. Louis, MO, USA) and a stable pool was used for experiments.

### 2.2. Viability Assay

SH-SY5Y cells were plated and treated with PPA (0.1, 1, 2, 6, and 12 mM, Sigma, St. Louis, MO, USA) in 96-well plates for 48 and 72 h Approximately 10 μL of CCK-8 (Dojindo, Kumamoto, Japan) was added to cells, and the optical density (OD) value was measured at 450 nm.

### 2.3. Immunocytochemistry

SH-SY5Y cells were grown on coverslips and treated with 1 mM PPA (Sigma, USA) for 72 h Cells were fixed with 1% paraformaldehyde (EMS, Hatfield, PA, USA) in phosphate-buffered saline (PBS, Welgene, Gyeongsangbuk-do, Gyeongsan-si, Korea) containing 4% sucrose for 5 min at room temperature. Primary antibodies against LC3A/B (#12741, Cell Signaling, Danvers, MA, USA) were added with blocking solution containing 0.1% gelatin, 0.3% Triton X-100, 16 mM sodium phosphate, and 450 mM NaCl, and cells were incubated overnight at 4 °C. After being washed with PBS, coverslips were incubated with Alexa Fluor488 (#4412, Cell Signaling, USA)-conjugated secondary antibodies for 1 h at room temperature and then again washed with PBS. Next, coverslips were mounted with a mounting medium (H-1000, Vector Laboratories, Burlingame, CA, USA) and were imaged with fluorescence microscopy (Nikon, Tokyo, Japan) using a 488 nm fluorescence filter.

### 2.4. Transmission Electron Microscopy for Quantifying Autophagic Elements

SH-SY5Y cells were treated with 1 mM of PPA for 72 h and then fixed with 2.5% glutaraldehyde/2% paraformaldehyde solution for 2 h Fixed cells were then post-fixed with 2% osmium tetroxide (EMS, USA) for 2 h at 4 °C, and the block was stained in 0.1 mg thiocarbohydrazide (TCH, TCI, Tokyo, Japan) in 10 mL distilled water and 1% uranyl acetate (EMS, USA) and dehydrated with a graded ethanol series. The samples were then embedded with an EMBed-812 embedding kit (EMS, USA). The embedded samples were sectioned (60 nm) with an ultramicrotome (Leica, Wetzlar, Germany), and the sections were then viewed on a Tecnai G2 transmission electron microscope (Thermofisher) at 120 kV. The numbers of autophagosomes and autolysosomes per cell were assessed.

### 2.5. Correlative Light and Electron Microscopy

CLEM was performed as previously described [23]. The mCherry–GFP tag fused to FIS1 stable SH-SY5Y cells were grown in 35 mm glass grid-bottomed culture dishes to 50–60% confluency. Cells with or without 1 mM or 2 mM PPA treatment were stained with 100 nM LysoTracker (LysoTracker Blue DND-22, Thermofisher, USA) for 15 min and then imaged under a confocal light microscope (Ti-RCP, Nikon, Japan), and after 24 h treatment of PPA, cells were fixed with 1% glutaraldehyde and 1% paraformaldehyde in 0.1 M cacodylate solution (pH 7.0). After being washed, cells were dehydrated with a graded ethanol series and infiltrated with an embedding medium. After embedment, 60 nm sections were cut horizontally to the plane of the block (UC7; Leica Microsystems, Germany) and were mounted on copper slot grids with a specimen support film. Sections were stained with uranyl acetate and lead citrate. The cells were observed at 120 kV in a Tecnai G2 microscope (ThermoFisher, USA). Confocal micrographs were produced as high-quality large images using PhotoZoom Pro 8 software (Benvista Ltd., Houston, TX, USA). Enlarged fluorescence images were fitted to the electron micrographs using the Image J BigWarp program.

### 2.6. Measurement of Mitophagy

The mCherry–GFP tag fused to FIS1 stable SH-SY5Y cells (5 × 10^4^ cells/well) was grown in 35 mm glass-bottomed culture dishes (MatTEK, Ashland, MA, USA) and treated with 1 and 2 mM PPA. Parallel incubation of cells without PPA was used for control. Measurement of mitophagy has been described previously [16]. Briefly, quantitation was performed for five fields of view for each group. Red-alone puncta were defined as round structures found only in the red channel with no corresponding structure in the green channel. Quantitative data were collected by manually counting all red-only puncta within each cell for each field of view.

## 3. Results

**Figure 1:**
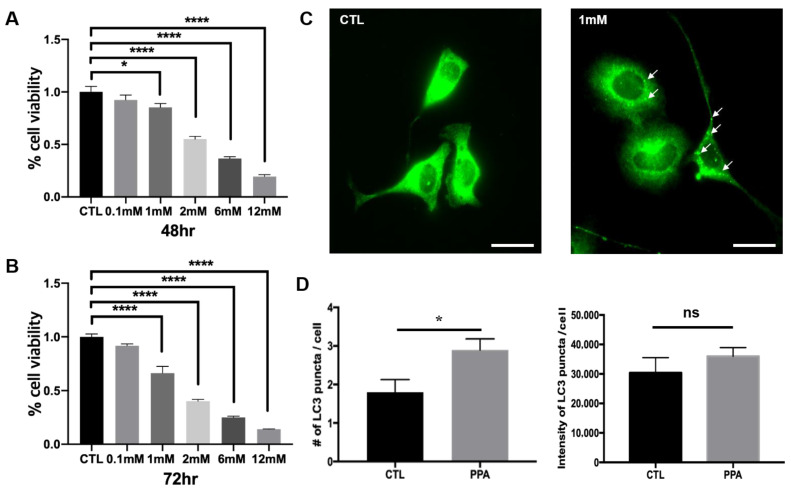
Viability assay and increase of of LC3 level in SH-SY5Y cells after propionic acid (PPA) treatment. The cells were treated with PPA for (**A**) 48 h and (**B**) 72 h Statistical analysis used a two- way ANOVA. Results are presented as mean ± SEM. When the concentration reached 2–12 mM, cell viability was significantly reduced. (**C**) Representative immunofluorescence images showing LC3 puncta in SH-SY5Y control cells and PPA-treated cells. The white scale bar is 50 µm. (**D**) Numbers of LC3 puncta and LC3 puncta intensity from images in (**A**) (n = 5), illustrating a significant increase of number of LC3 puncta following treatment with 1 mM PPA. Statistical analysis used a one-way ANOVA. Results are presented as mean ± SEM. * *p* < 0.05, **** *p* < 0.0001.

The viability of SH-SY5Y cells after treatment with PPA showed that optimal concentrations of PPA were less than 2 mM. Viability was assessed with CCK-8 assays after treatment with concentrations of 0, 0.1, 1, 2, 6, and 12 mM. Viability was significantly decreased in response to 2 mM after 48 h incubation with PPA (Figure 1A). After 72 h, treatment with 1 mM PPA a significant change was also shown (Figure 1B). Therefore, we used 1 mM PPA for 72 h to assess autophagy in SH-SY5Y cells. The number of LC3 puncta in PPA-treated cells was increased, as shown by immunofluorescence (Figure 1C). The number of LC3 puncta was 2.9 ± 0.28, compared with 1.8 ± 0.3293 for untreated cells (Figure 1D). No difference in the intensity of LC3 puncta was observed.

**Figure 2:**
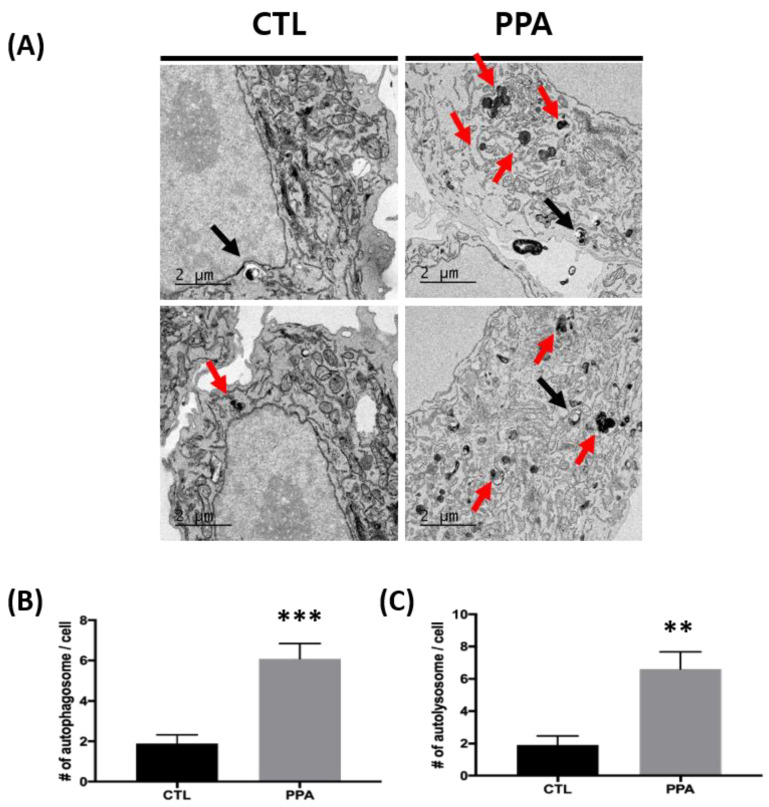
The increase in autophagy following PPA treatment in SH-SY5Y cells using TEM. (**A**) Representative TEM images showing autophagy in control SH-SY5Y cells and PPA-treated cells. The black arrow indicates autophagosomes, and red arrows indicate autolysosomes. (**B** and **C**) Numbers of autophagosomes and autolysosomes from images in (**A**) (n = 10). TEM analysis shows that numbers of both autophagosomes and autolysosomes increase in cells treated with 1 mM PPA. Statistical analysis used a one-way ANOVA. Results are presented as mean ± SEM. ** *p* < 0.01, *** *p* < 0.0005.

We further analyzed the number of autophagosomes and autolysosomes in control and PPA-treated cells using TEM images. The numbers of both organelles were increased in PPA-treated cells. The numbers of autophagosomes per cell were 6.1 ± 0.7371 and 1.9 ± 0.4333 for treated and control cells, respectively (Figure 2A). Similar results for autolysosomes were found: 6.6 ± 1.067 for PPA-treated cells and 1.9 ± 0.5667 for untreated cells (Figure 2B).

**Figure 3:**
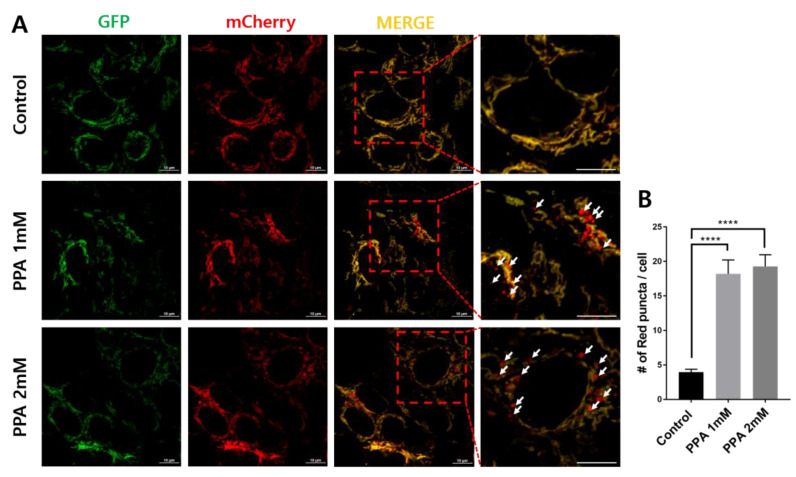
Mitophagy assays in SH-SY5Y cells following treatment with 1 and 2 mM PPA for 24 h. (**A**) mCherry–GFP-FIS1101–152 stably expressing cells were subjected to (1) control, (2) 1 mM PPA, and (3) 2 mM PPA for 24 h. (**B**) Numbers of red puncta (mitophagy) per cell (n > 40). Statistical analysis used a one-way ANOVA. Results are presented as mean ± SEM. **** *p* < 0.0001.

PPA was reported as a small molecule leading to mitochondrial dysfunction [7]. Therefore, we used the tandem mCherry–GFP tag fused to FIS1-stable SH-SY5Y cells to confirm the induction of mitophagy by PPA treatment. GFP and mCherry show green and red fluorescence, respectively, with the former specific for mitochondria and the latter for mitophagy (Figure 3A). After treatment with 1 or 2 mM PPA, the number of mCherry red puncta increased 4.6 times, indicating the induction of mitophagy in treated cells (Figure 3B).

**Figure 4:**
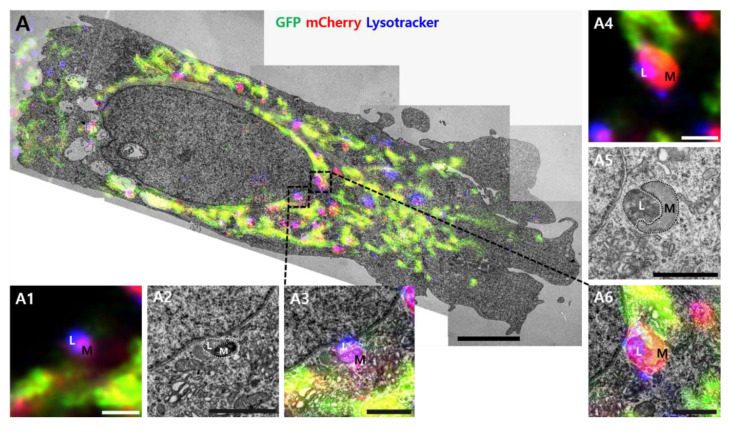

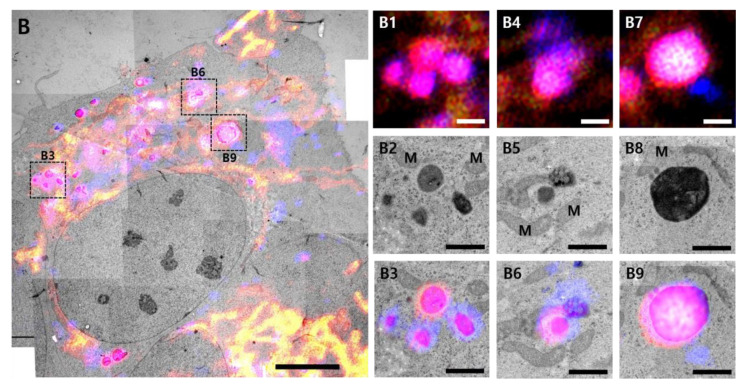
Correlative light and electron microscopy. Correlative confocal and electron microscope images of (**A**) co-localized mCherry–GFP-FIS1101–152 in SH-SY5Y cells or (**B**) cells treated with 2 mM PPA. Yellow indicates co-localization of GFP and mCherry signals. Magenta indicates co-localization of mCherry and LysoTracker. Multiple TEM images were taken at 1700× magnification. Images were stitched for a large field of view at higher resolution. The black dot line box indicates structures corresponding to magenta fluorescent puncta on fluorescence images. The structures showed the morphology of mitophagy, as demonstrated by the black dot line box shown at higher magnification in the inserted images (A1–A6 and B1–B9). L, lysosome; M, mitochondria. Size bar in A and B = 5 µm, A1~A6 = 1 µm, B1~B9 = 1 µm.

We employed CLEM to confirm the ultrastructure of red puncta (Figure 4). Live cells were imaged using confocal microscopy treatment with PPA after 24 h. Images were then aligned with stitched TEM images of the same cells. Almost healthy mitochondria showed the green fluorescence of GFP, and some red puncta were co-localized with LysoTracker (Figure 4A). In control cells, LysoTracker-positive structures (blue) are seen contacting red puncta (Figure 4A, white dot line box, and enlarged images). In the PPA treatment cells, damaged round mitochondria show high electron density in the electron micrographs and are co-localized with LysoTracker, suggesting mitophagy (Figure 4B).

## 4. Discussion

Mitochondria are continuously replenished. As new mitochondria are produced, dysfunctional organelles are removed by autophagy-mediated degradation though mitophagy [24]. There are several control or repair systems for mitochondrial structure and function maintaining essential energy metabolism. Oxidatively damaged proteins in the mitochondrial outer membrane can be degraded by the ubiquitin–proteasome system. When damage is more extensive, e.g., through exposure to elevated reactive oxygen species (ROS) or aging, mitochondria are sequestered by autophagosome and fused with lysosome for degradation. It is called mitophagy. The extent of mitophagy in neurites is influenced by various factors related to mitochondrial function, and the contribution of mitophagy to mitochondrial function in soma or neurites is critical to understanding the regulation of energy metabolism in these cells [25]. Our previous work shows that PPA induces defects in mitochondria [6] and autophagy [10]. In this study, we investigated the relationship between mitochondrial dysfunction and increased autophagy.

Some mitochondrial toxins, including rotenone, concomitantly activate autophagy, including mitophagy. Like rotenone, PPA-treated cells showed elevated autophagic sequestration of mitochondria. More prominent LC3 signals (Figure 1C) and LC3-II/β-actin ratios (data not shown) indicate change of autophagy in PPA-treated cells. Several reports that focus on mitochondrial function in PPA-treated cells are available. Kim et al. showed an increase of mitochondrial copy number and expression of PGC-1a, COX4, SIRT3, and, TFAM (mitochondrial biogenesis-related proteins) after PPA treatment of SH-SY5Y cells [7]. Dysfunction of mitochondria caused by RNA interference-mediated knockdown of peroxisome proliferator-activated receptor-γ coactivator 1α (PGC-1α) in neurons showed abnormal synapse formation in developing neural circuits and failure to maintain synapses in the hippocampus of adults [26]. Induction of mitochondrial biogenesis following expression of PGC-1α is stimulated by brain-derived neurotrophic factor, which can be modulated by changes of SCFAs in the brain [27]. PPA-induced mitochondrial dysfunction suggests a mechanism for neurotoxicity. El-Ansary et al. showed PPA-induced neurotoxicity in rat pups though depletion of gamma-aminobutyric acid and serotonin. [28] Frye et al. showed oxidative stress after PPA exposure, and Alfawaz et al. showed that factors related to mitochondria, such as carnosine, N-acetylcysteine, and vitamin D, can rescue neurotoxicity caused by PPA in rat pups [29]. The protective effect of carnosine (β-alanyl-L-histidine) is related to autophagy and causes a decrease in Drp-1 expression. Further, treatment with N-acetylcysteine shows inhibition of Atg32-dependent mitophagy [29]. In PPA-treated SH-SY5Y cells in the present study, TEM analysis shows an increase in numbers of both autophagosomes and autolysosomes, which reflects properly functioning autophagy flux (Figure 2).

Several technical challenges using fluorescence and biochemical techniques to analyze autophagic processes, including mitophagy, are recognized [17,30]. Such challenges were met in this study using CLEM techniques to monitor autophagy for cellular homeostasis due to mitochondrial dysfunction in PPA-treated SH-SY5Y cells. The tandem mCherry–GFP tag fused to FIS1 was used in our approach. Red fluorescence of mCherry increased in PPA-treated cells, suggesting increased mitophagy, and the green fluorescent of GFP in mitochondria did not change significantly (Figure 3). CLEM confirmed the ultrastructure associated with these colors as mitochondria and mitophagy (Figure 4). There is a mismatch between some healthy mitochondrial fluorescence signal (yellow color) and EM images due to technical limitations of the TEM based CLEM method (Supplementary Figure S1 A1 and B1). It is difficult to accurately match the Z axis of the optical section (LM) and the physical section (EM), since LM and EM image thicknesses are different. Due to the difference, there is more information in the fluorescence micrograph (LM: 300 nm optical thickness, EM: 60 nm physical thickness). Although there is some technical limitation, the damaged mitochondria are well correlated with the lysotracker (Supplementary Figure S1, black arrow). Thus, observations depicted in Figure 3 and Figure 4 indicate that increased autophagy shown in Figure 1 and Figure 2 is mitophagy.

## 5. Conclusions

Changes in mitophagy under stress condition is associated with pathological conditions, including neurodegenerative diseases and myopathies. Therefore, identifying mitophagy modulators and understanding their mechanisms of action will provide critical insight into neurodegenerative diseases. We confirmed that mitophagy was induced by PPA treatment in SH-SY5Y cells. CLEM is a useful technique for monitoring mitophagy in cells under stress. Various stages of mitophagy, including initiation of autophagy, vesicle completion, lysosome fusion, and degradation of mitochondria in lysosomes, can be monitored in CLEM, if the time points in such studies are adequately controlled. CLEM might also be applied to study structural changes of other cellular organelles.

## Supplementary Materials

The following are available online at https://www.mdpi.com/1996-1944/13/19/4336/s1, Figure S1: Correlative confocal and electron microscope images of the control (A) and 2 mM PPA treated cell (B).
