# Supplementary material for: Correlative Light and Transmission Electron Microscopy Showed Details of Mitophagy by Mitochondria Quality Control in Propionic Acid Treated SH-SY5Y Cell
Source: Materials (Basel). 2020 Sep 29;13(19):4336. doi: 10.3390/ma13194336 (PMC7579125; doi:10.3390/ma13194336)
Supplement: Supplementary file 1 [file materials-13-04336-s001.pdf]

Supplementary

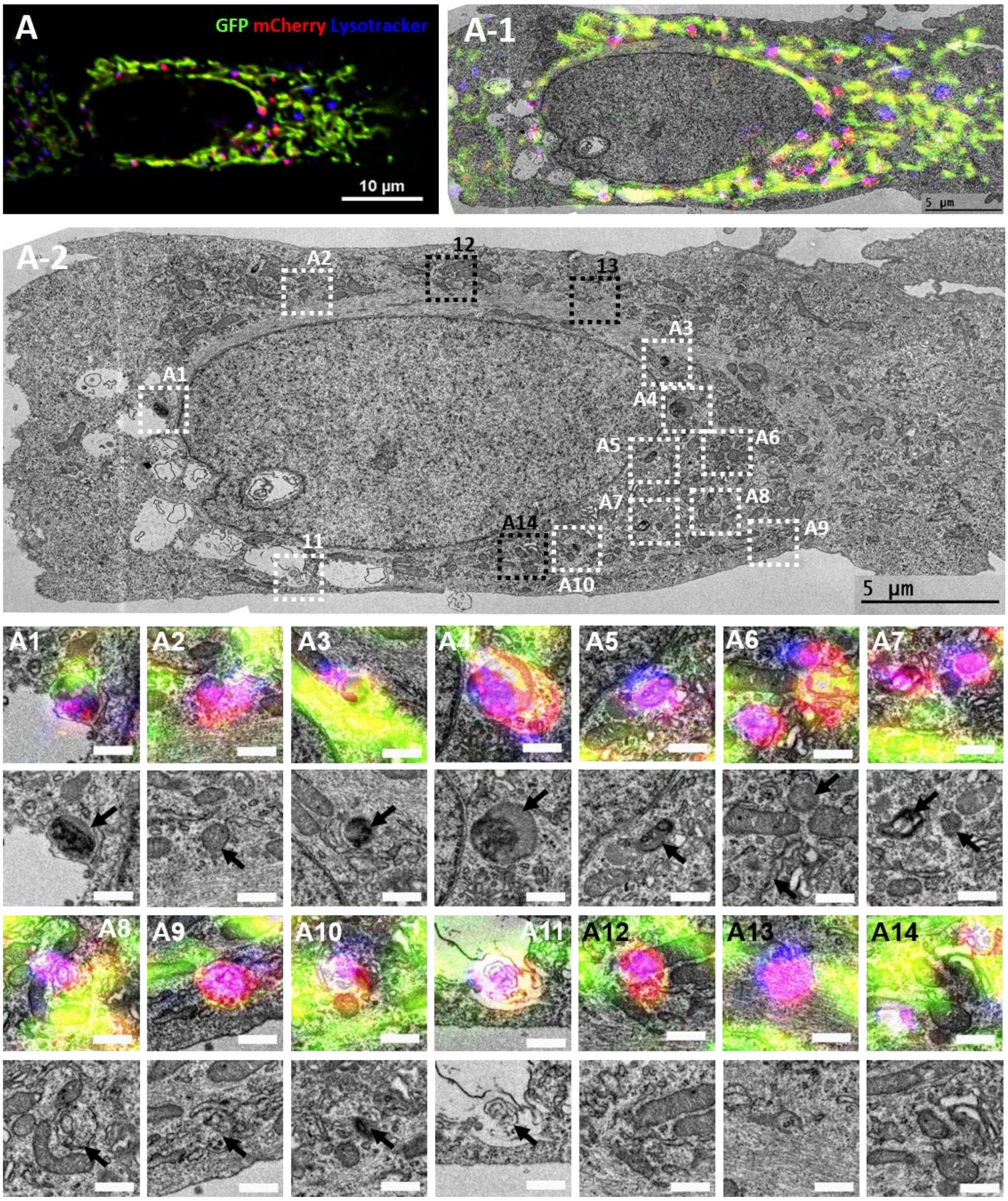

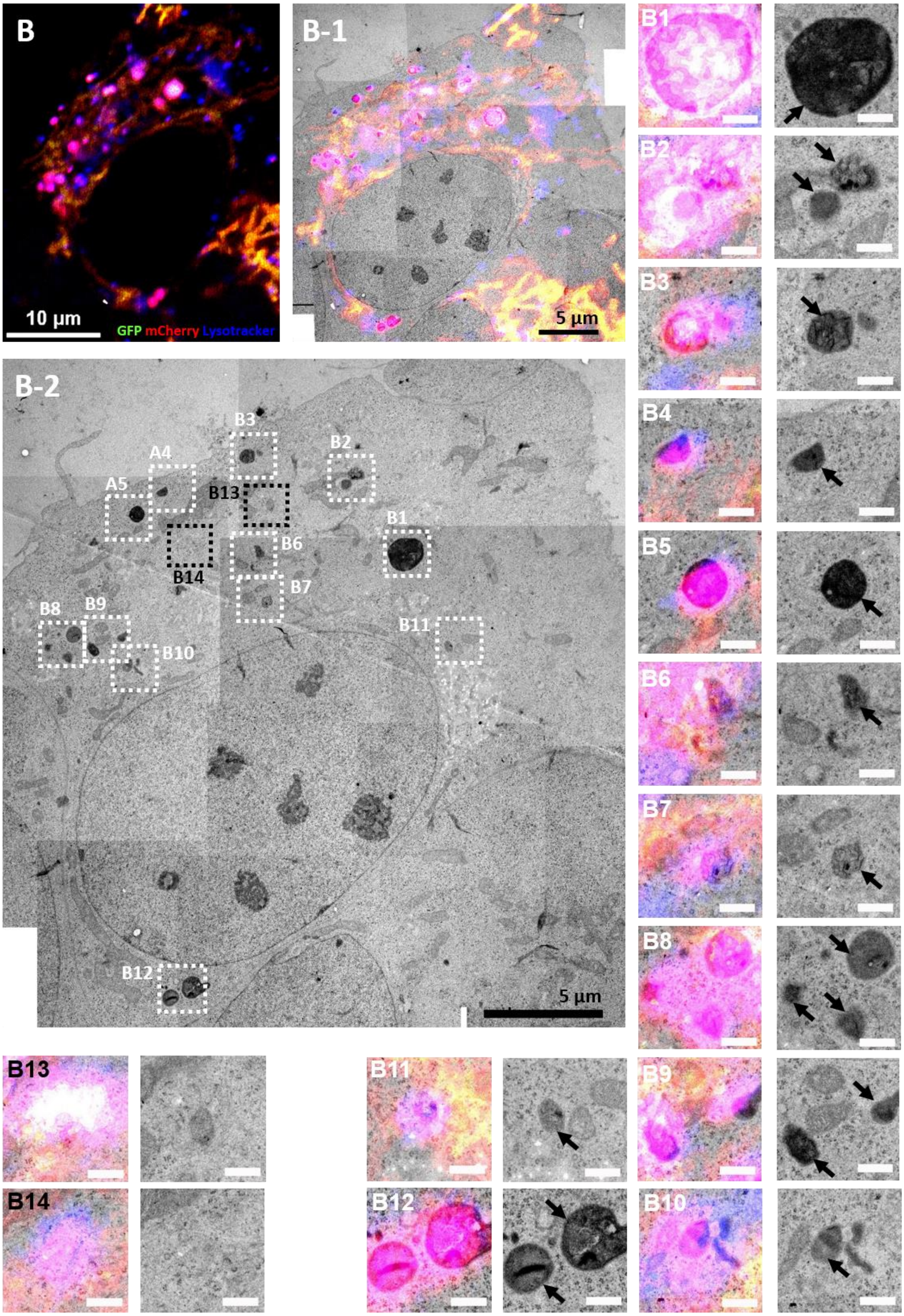

Figure S1. Correlative confocal and electron microscope images of the control (**A**) and 2 mM PPA treated cell (**B**).

(Figure S1A,B) Live cell imaging of SH-SY5Y cells stably expressing mCherry–GFP tag fused to FIS1. Healthy mitochondria and mitophagy are visualized in the yellow and pink color, respectively. The lysotracker is shown in the blue channel. (A-1 and B-1) Composite image of the fluorescence and electron micrograph. (A-2 and B-2) Electron microscope image for observation of the subcellular organelle ultrastructure. Almost pink fluorescence signals and mitophagic ultrastructures were well correlated (A1~A11 and B1~B12), But some region is not exactly correlated (A12~14 and B13~B14). Black arrow = mitophagy, Size bar in 1~14 = 500 nm.
